# Supplementary material for: A mutual information based R-vine copula strategy to estimate VaR in high frequency stock market data
Source: PLoS One. 2021 Jun 17;16(6):e0253307. doi: 10.1371/journal.pone.0253307 (PMC8211166; doi:10.1371/journal.pone.0253307)
Supplement: Attachment — Submitted filename: Response to Reviewers.docx [file pone.0253307.s002.docx]

Dear Editor,

Our sincere thanks to you and all the reviewers for your valuable comments on the scientific content and presentation of our manuscript titled “A Mutual Information Based R-Vine Copula Strategy To Estimate VaR in High Frequency Stock Market Data” **(PONE-D-20-23135)**. We have made several changes to manuscript in accordance with these comments.

We hope that the revised version of the manuscript will be considered favourably by PLOS ONE. We now give an item wise response to the reviewers’ comments:

**Regarding Journal Requirements:**

*1) Please ensure that your manuscript meets PLOS ONE's style requirements, including those for file naming.*

We crosschecked and the revised manuscript is in accord with the requirements.

*2) Restriction on sharing data*

We have uploaded the processed data as a supporting file “S1 Table”. The raw data is “available from a third party”, details of which are provided in the cover letter as well: NSE Data & Analytics (<https://www.nseindia.com/supra_global/content/dotex/about_dotex.htm>). . The terms of purchase prohibit us from redistributing the Historical Data or any component of it.

Revised Data Availability Statement:

Raw data cannot be shared publicly because of copyright agreement with NSE Data & Analytics, formerly known DotEx International Ltd.(third party). Data are available from the NSE Data & Analytics, formerly known DotEx International Ltd., (contact via dotex_kraops@nse.co.in) for researchers who meet the criteria for access to confidential data.

The terms of purchase prohibit us from redistributing the Historical Data or any component of it. However, the raw data can be purchased from NSE Data & Analytics at: https://www.nseindia.com/supra_global/content/dotex/data_products.htm

We also confirm that we did not have any special access privileges that others would not have.

However, we also confirm that all the processed data corresponding to each figure and each table is uploaded as a supporting file named “S1 Table”.

*3) Please review your reference list to ensure that it is complete and correct. If you have cited papers that have been retracted, please include the rationale for doing so in the manuscript text, or remove these references and replace them with relevant current references. Any changes to the reference list should be mentioned in the rebuttal letter that accompanies your revised manuscript. If you need to cite a retracted article, indicate the article’s retracted status in the References list and also include a citation and full reference for the retraction notice.*

While adhering to the editor’s suggestion of including a brief description of ARCH and GARCH, we needed to include a fresh reference of a paper by Robert F. Engle. The list of references have been revised in view of the stated changes and we are certain that no references correspond to retracted manuscripts. Further, we have updated the missing DOIs and ISBN(in case of books) information as well. All the relevant changes have been incorporated in the revised document.

**Additional Editor Comments:**

1)*I would suggest a brief description of ARMA and GARCH models and the meaning of their parameters. How did you check the fit of these models, just using AIC?*

Specific details about ARCH and GARCH models have been inserted in section 3.1(lines 271-279) of the manuscript.

Fitting of the three models i.e. GARCH(1,1), ARMA(1,1)-GARCH(1,1) and ARMA(1,1)-EGARCH(1,1) were based on the two steps. First step: p-values for each parameter in each model was observed to be significantly high justifying the fit of the respective models. Second step: to pick the best model out of the three we used minimum AIC criteria.

2)*For the univariate distribution, you explained you considered both NIG and Student's t and you selected the latter due to its simplicity. What do you specifically mean? That it is easier to estimate its parameters? How is Kolmogorov-Smirnov test affected by the fact that parameters are unknown and need to be estimated? As far as I know, estimating unknown parameters may lead to a "biased" p-value. Did you explored other parametric families (eg stable distributions)?*

NIG is a subclass of generalized hyperbolic distribution depending upon 4 parameters. On the other hand, Student’s t-distribution depends only on 1 parameter. Considering errors associated with the estimation of the parameters, it is always beneficial to estimate one parameter over 4 parameters. We tried fitting of both the distributions on the univariate data and ran Kolmogrov Smirnov test to check goodness of fit. All 50 stocks cleared the test at 1% level of significance in both the cases. To keep our computational errors low we decided to pick Student’s t-distribution over NIG.

Kolmogrov-Smirnov test was conducted using simulations, which ensured unbiased p-values.

Since NIG and t-distribution gave us satisfying results, thus we decided to stick to them and didn’t explore further.

3)*Figures are blurred, please fix them*

We have re-generated all figures at 600dpi and ran through the PACE software. Hope they are of acceptable quality now.

4)*Perhaps when explaining the application of vines some graphs could be beneficial to a better understanding*

Thank you for your valuable suggestion. We have now included an example in section 3.1(refer to lines 204-213 of the manuscript) along with the associated graph (Fig 1).

**Reviewer #1:**

1)*…one minor suggestion. The authors repeated refer to "mutual information," which is not a phrase with which I'm familiar. I believe they mean "dependence," in the statistical sense. Could they more clearly define (very early in the paper) what they mean by "mutual information"?*

Thanks for your valuable suggestion. We have now included the definition of mutual information in the introduction section 1 (lines 69-74) and explained it in detail in section 3.2(lines 232-244).
